# Single-Nucleotide Polymorphisms in *LPA* Explain Most of the Ancestry-Specific Variation in Lp(a) Levels in African Americans

**DOI:** 10.1371/journal.pone.0014581

**Published:** 2011-01-24

**Authors:** Rahul C. Deo, James G. Wilson, Chao Xing, Kim Lawson, W. H. Linda Kao, David Reich, Arti Tandon, Ermeg Akylbekova, Nick Patterson, Thomas H. Mosley, Eric Boerwinkle, Herman A. Taylor

**Affiliations:** 1 Department of Genetics, Harvard Medical School, Boston, Massachusetts, United States of America; 2 Cardiology Division, Massachusetts General Hospital, Harvard Medical School, Boston, Massachusetts, United States of America; 3 Department of Medicine, Harvard Medical School, Boston, Massachusetts, United States of America; 4 Department of Physiology and Biophysics, University of Mississippi Medical Center, Jackson, Mississippi, United States of America; 5 Department of Medicine, University of Mississippi Medical Center, Jackson, Mississippi, United States of America; 6 Donald W. Reynolds Cardiovascular Clinical Research Center, The Eugene McDermott Center for Human Growth and Development, University of Texas Southwestern Medical Center at Dallas, Dallas, Texas, United States of America; 7 Human Genetics Center and Institute of Molecular Medicine, University of Texas Health Science Center at Houston, Houston, Texas, United States of America; 8 Department of Epidemiology, Johns Hopkins Bloomberg School of Public Health, Baltimore, Maryland, United States of America; 9 Program in Medical and Population Genetics, Broad Institute, Cambridge, Massachusetts, United States of America; 10 Jackson State University, Jackson, Mississippi, United States of America; 11 Tougaloo College, Tougaloo, Mississippi, United States of America; University of Chicago Howard Hughes Medical Institute, United States of America

## Abstract

Lipoprotein(a) (Lp(a)) is an important causal cardiovascular risk factor, with serum Lp(a) levels predicting atherosclerotic heart disease and genetic determinants of Lp(a) levels showing association with myocardial infarction. Lp(a) levels vary widely between populations, with African-derived populations having nearly 2-fold higher Lp(a) levels than European Americans. We investigated the genetic basis of this difference in 4464 African Americans from the Jackson Heart Study (JHS) using a panel of up to 1447 ancestry informative markers, allowing us to accurately estimate the African ancestry proportion of each individual at each position in the genome. In an unbiased genome-wide admixture scan for frequency-differentiated genetic determinants of Lp(a) level, we found a convincing peak (LOD = 13.6) at 6q25.3, which spans the *LPA* locus. Dense fine-mapping of the *LPA* locus identified a number of strongly associated, common biallelic SNPs, a subset of which can account for up to 7% of the variation in Lp(a) level, as well as >70% of the African-European population differences in Lp(a) level. We replicated the association of the most strongly associated SNP, rs9457951 (p = 6×10^−22^, 27% change in Lp(a) per allele, ∼5% of Lp(a) variance explained in JHS), in 1,726 African Americans from the Dallas Heart Study and found an even stronger association after adjustment for the kringle(IV) repeat copy number. Despite the strong association with Lp(a) levels, we find no association of any *LPA* SNP with incident coronary heart disease in 3,225 African Americans from the Atherosclerosis Risk in Communities Study.

## Introduction

Lipoprotein(a) (Lp(a)) is a subclass of lipoproteins, consisting of a low-density lipoprotein (LDL)-like particle covalently bound to the *LPA* gene product. Serum Lp(a) levels are a risk factor for cardiovascular disease, albeit with more modest effect than LDL-cholesterol [1]–[3]. Common variants within the *LPA* gene have been associated with myocardial infarction, suggesting a causal link between Lp(a) and atherosclerotic heart disease [4]–[7].

The genetic determinants of Lp(a) levels have been investigated extensively both within and between ethnic groups. Lp(a) is highly variable, with over 90% of the variance in Lp(a) levels in European Americans attributable to variation within the *LPA* gene [4]; the corresponding percentage in African Americans is ∼80% [5]. *LPA* includes a well-characterized 5.6 kilobase-pair copy-number variant (CNV) that encodes a kringle(IV) domain [6], [7]. Higher copy numbers for this domain are associated with lower Lp(a) levels [8], presumably due to impaired secretion of the larger protein product [9]. Biallelic SNPs and other CNVs also appear to contribute independently to Lp(a) level [10].

Lp(a) levels vary widely between populations [11], with some populations of African ancestry having nearly 4-fold higher Lp(a) levels than European Americans [12]. The interethnic differences in populations seem to be only weakly explained by the kringle(IV) CNV, thus motivating searches for other responsible variants [10]. Given the association of *LPA* variants with coronary heart disease (CHD), identifying these determinants may have clinical implications for differences in disease prevalence between populations.

Recent genetic association analyses in admixed populations such as African Americans have highlighted the complexities due to confounding by ancestry [13]. We have extended our earlier work on admixture mapping and genetic association analysis in the Jackson Heart Study (JHS) to the Lp(a) trait and find the amount of African or European ancestry at the *LPA* locus is strongly associated with Lp(a) level. Dense fine-mapping of *LPA* identified multiple strongly associated variants, including rs9457951 and rs10455872, a SNP strongly associated with myocardial infarction in European populations [14]. A multi-SNP model explains ∼7% of the variation in Lp(a) level and 73% of the association of local ancestry with this trait. We have replicated the strongest association (rs9457951) in the Dallas Heart Study (DHS), and find a stronger effect after adjustment for the kringle(IV) CNV. Finally, we genotyped 10 SNPs in >3200 African Americans in the Atherosclerosis Risk in Communities (ARIC) Study and, although we validated the strong association of *LPA* local ancestry and genotypes at 7 of the 10 SNPs with Lp(a) level, we find no significant association of these variables with incident CHD.

## Results

### Admixture Mapping for Determinants of Lp(a) Levels Identifies a Strong Peak at the *LPA* Locus

We studied a sample of 4605 individuals from the JHS (Table 1), a community-based observational study of cardiovascular disease (CVD) in African Americans [15]. We have previously used a panel of >1400 genotypic markers selected for high differences in frequency between European Americans and West Africans [16] to estimate African ancestry across the genome of 4464 individuals. To investigate the genetic basis of African-European differences in serum Lp(a) levels, we performed genomewide admixture mapping of the Lp(a) trait (see Methods).

**Table 1:** Demographic Characteristics for the Genotyped Jackson Heart Study Participants.

	All	Unrelated	Unrelated with only African local ancestry at *LPA*	Unrelated with European local ancestry at *LPA*
Number	4464	3300	1831	615
Male (%)	36.8	37.8	38.4	40.7
Age (mean - years, ± std.)	55±13	56±11	57±12	57±12
BMI (mean - kg/m^2^ ± std.)	32±7	32±7	32±7	31±6
Type II DM (%)	18.9	20.4	20.6	17.6
Cholesterol or TG-Lowering Medication (%)	12.5	13.6	13.8	14.3
LDL-Cholesterol: unmedicated | medicated participants (mg/dL)	127±36 | 113±33	128±37 | 113±33	128±36 | 113±35	130±38 | 117±30
HDL-Cholesterol: unmedicated | medicated participants (mg/dL)	51±14 | 51±14	52±15 | 52±14	52±15 | 51±14	52±14 | 52±12
Serum Triglycerides: unmedicated | medicated participants (mg/dL)	106±81 | 129±151	107±83 | 130±164	108±97 | 136±212	108±63 | 123±73
Serum Lp(a): unmedicated | medicated participants (mg/dL)	57±44 | 72±54	57±43 | 73±54	62±43 | 83±57	42±37 | 55±43
Overall African Ancestry (%)	83±9	82±9	85±7	75±12

Characteristics are shown for the 4464 genotyped participants, the 3300 unrelated individuals, the 1831 unrelated individuals with homozygous African local ancestry at the *LPA* locus, and the 615 individuals with at least one ancestral European allele at *LPA*.

**Figure 1:**
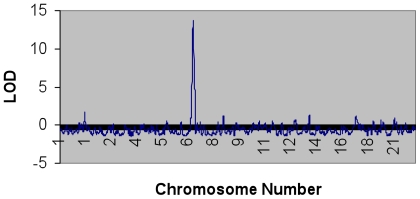
Affected-Only Statistic at Equally Spaced Points across the genome for the Lp(a) Admixture Scan. The 95% credible interval for the peak at 6q25.3 includes the *LPA* and *LPA2* genes.

Admixture mapping of Lp(a) reveals a compelling association of increased African ancestry with Lp(a) case status (upper quintile) at chromosome 6q25.3 (LOD 13.6, Figure 1). This far exceeds our threshold of significance of 5 for LOD scores [17] and suggests a marked association of local ancestry at this locus with Lp(a) level. In this region, individuals having Lp(a) levels in the upper quintile had a mean African ancestry of 87.4%, compared to 72.7% for those having Lp(a) values in the lower quintile (p<2×10^−16^). The 95% credible interval for this peak spans from 158 to 162 megabasepairs (Mb) and includes the *LPA* gene.

### Local Ancestry at the *LPA* locus is strongly associated with Lp(a) Levels

In addition to localizing genetic determinants of disease, estimates of individual ancestry in admixed individuals can be correlated with continuous phenotypes (see Methods). To further characterize the admixture peak at the *LPA* locus, we obtained an estimate for overall ancestry and for local ancestry at the *LPA* locus for each individual in JHS. Looking first at overall ancestry, we found a 9.9±1.3% increase in Lp(a) level for each 10% increase in overall African ancestry (p = 6.8×10^−7^). Interestingly, this result is in keeping with the ∼2 fold difference in Lp(a) levels seen in ARIC between European and African Americans [18], suggesting that genetic determinants of the association between global ancestry and Lp(a) levels within an admixed population may prove useful in explaining differences in Lp(a) levels between African and European American populations. When we incorporated local ancestry as a covariate in the linear regression model, we found an increase of 7.7±0.5% in Lp(a) level for each 10% increase in local African ancestry at the *LPA* locus (p = 1.8×10^−25^). Furthermore, inclusion of the *LPA* local ancestry rendered the overall ancestry term non-significant, suggesting that local ancestry at *LPA* almost fully explains the ancestry-related differences in Lp(a) levels.

### Common *LPA* SNPs Explain A Modest Percentage of Variance in Lp(a) Levels

We next performed a fine-mapping study to look for variant(s) in the *LPA* gene that can account for the admixture signal at this locus. Although several common variants as well as the kringle(IV) repeat have previously been shown to contribute to African-European differences [10], these earlier studies were performed without estimates of *LPA* local ancestry. In such cases, effects attributed to the repeat or to specific SNPs may reflect confounding by admixture linkage disequilibrium.

Given that genetic variation at the *LPA* locus has been shown to explain >80% of variation in Lp(a) levels [5], we selected a dense panel of SNPs spanning the *LPA* locus and extending 10 kb upstream and downstream (see Methods). This interval includes *LPA* and nearly all of *LPAL2* (Lp(a)-like 2 precursor). SNP-Lp(a) associations were evaluated by linear regression, correcting for global and local ancestry at the *LPA* locus. As another approach to potential confounding by differing LD patterns, we tested association of each SNP separately in a subpopulation of 1831 individuals who had >95% probability of two African ancestral chromosomes in the region (JHS-AFR-2*_LPA_*), thus minimizing heterogeneity in local ancestry background, and a subpopulation of 615 individuals with >95% probability of at least one European ancestral allele (JHS-EUR-1_2*_LPA_*; see Table 1 for demographic characteristics of these subgroups).

**Figure 2:**
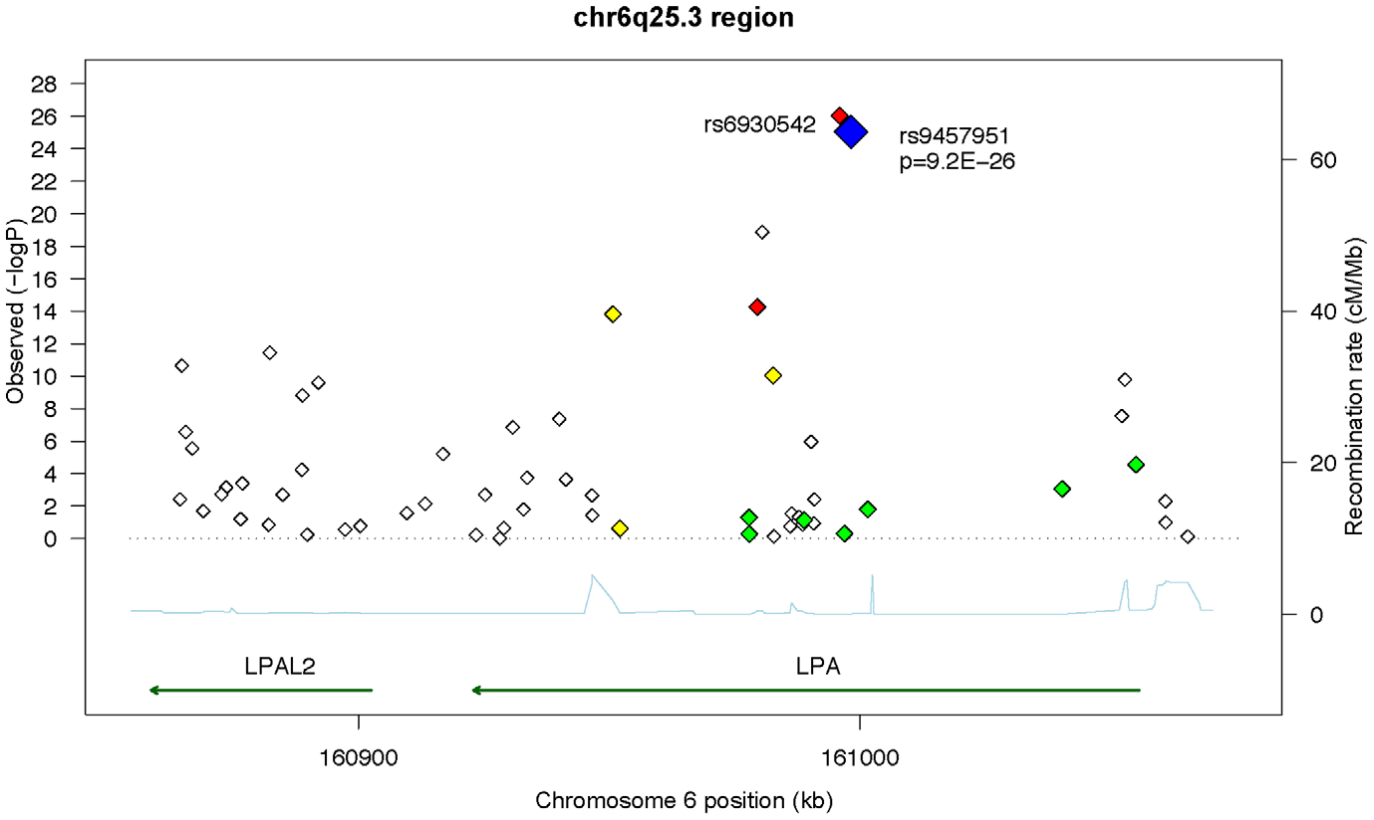
Graphical Depiction of SNP-Lp(a) Associations in the 6q25.3 region. For each SNP, -log(p-values) given in Table S1 are shown against chromosomal position (kb). rs9457951 is highlighted in blue, and SNPs in strong (r^2^≥0.8), moderate (0.8>r^2^≥0.25) and weak(0.25>r^2^≥0.10) and very weak (0.1>r^2^) linkage disequilibrium with rs9457951 in the Yoruba HapMap population are depicted by red, yellow, green, and white diamonds, respectively. The position of the *LPA* and *LPAL2* genes are depicted by green arrows. The chromosomal recombination rate for the Yoruba population is depicted at the bottom of the plot, in light blue.

**Figure 3:**
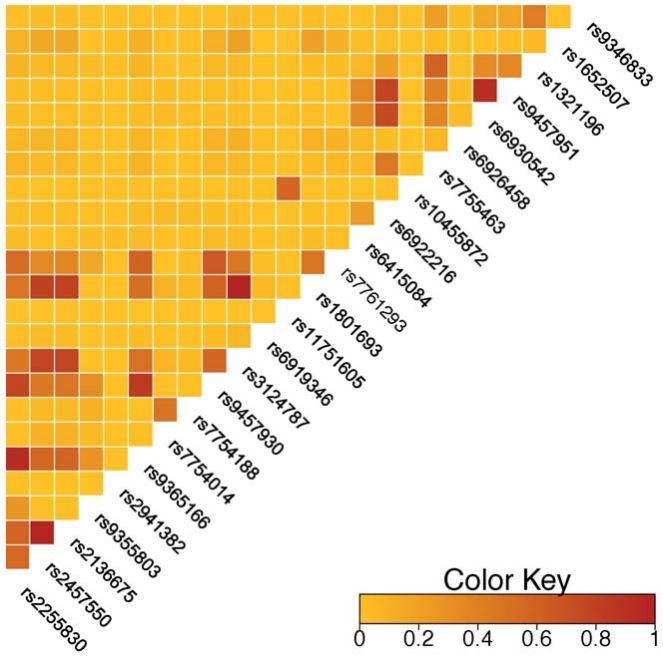
Pairwise linkage disequilibrium measures (r^2^) for significantly associated SNPs in the JHS African local ancestry subpopulation.

Figure 2 and Table S1 present the p-values for association with Lp(a) for the 59 successfully genotyped SNPs. 24 SNPs had a p-value less than 0.00085 (corresponding to p = 0.05 with Bonferroni correction for 59 SNPs tested), with the strongest SNPs being rs9457951 (p = 9.2×10^−26^), rs6930542 (p = 9.2×10^−27^, r^2^ = 0.994 with rs9457951 in JHS-AFR-2 *_LPA_*), rs10455872, (p = 1.3×10^−19^) and rs6922216 (p = 5.4×10^−15^, r^2^ = 0.746 with rs9457951 in JHS-AFR-2*_LPA_*). Eighteen of the top 20 SNPs show significant association in the JHS-AFR-2*_LPA_* subpopulation at p<0.00085. The exceptions were rs10455872, which is nearly fixed in frequency (MAF = 0.001) in JHS-AFR-2*_LPA_*, and rs6919346, which has an MAF of 0.006 in this subgroup. Notably, for 21 of the 24 significant SNPs, the allele associated with higher Lp(a) levels is at higher frequency in the African ancestral subpopulation, providing a strong explanation for the association of African *LPA* local ancestry with increased Lp(a) levels. Furthermore, for 4 of the SNPs – rs9457951, rs6930542, rs6922216, rs7755463 – the allele corresponding to higher Lp(a) levels is nearly absent in JHS-EUR-2*_LPA_*. Pairwise LD values in the African and European local ancestry subpopulations are shown in Figures 3 and Figure S1.

Effect sizes for individual SNPs are shown in Table 2. For rs9457951, which is intronic, the effect size is an increase in Lp(a) level by 25±2% per allele. The minor allele frequency for rs9457951 in JHS is 0.19, and this SNP alone appears to explain ∼5% of the residual variance in Lp(a) after adjustment for gender. The largest effect is seen for rs10455872, which demonstrates an effect size of 92±7% in Lp(a) level per inherited allele. This SNP is intronic, and was recently shown to explain ∼25% of the Lp(a) variance in a European cohort with a positive association with myocardial infarction [14]. The high Lp(a) rs10455872 genotype is correlated with smaller *LPA* isoform number and thus the strong contribution to Lp(a) variance likely arises from the effect of the well known kringle(IV) repeat in the *LPA* gene.

**Table 2:** Effect Size of *LPA* variants on Lp(a) levels.

SNP	Allele	f_all_	f_afr_	f_eur_	effect_all_ ± se_all_	effect_afr_ ±se_afr_	effect_eur_ ±se_eur_	p_int
rs2255830	T	0.776	0.847	0.422	14.5±2	16.5±3	6.3±4.3	0.126
rs2457550	T	0.872	0.918	0.727	13.8±2.5	16.5±3.8	1.4±4.9	0.0136
rs2136675	A	0.861	0.906	0.625	12.7±2.6	16.8±3.9	−0.4±4.9	6.4E-03
rs9355803	C	0.927	0.949	0.814	11.3±3.3	11.1±4.9	11.4±6	0.860
rs2941382	G	0.95	0.976	0.856	14.9±4	9±7.4	18.1±6.7	0.656
rs9365166	C	0.782	0.853	0.422	15.1±2	18.3±3	6.6±4.4	0.090
rs7754014	T	0.444	0.495	0.233	6.8±1.6	8.1±2.1	−6.8±4.1	1.9E-03
rs7754188	T	0.305	0.325	0.222	11.4±1.8	14±2.3	−0.1±4.4	6.6E-03
rs9457930	T	0.795	0.869	0.439	14.3±2.1	15.4±3.3	7±4.5	0.176
rs3124787	T	0.866	0.918	0.6	12.3±2.6	16.5±4.1	0.5±5	0.013
rs6919346	C	0.965	0.994	0.81	27.5±4.7	36.8±15.5	22.8±7	0.347
rs11751605	C	0.024	0.001	0.107	21.5±5.3	106.5±46.4	31.8±7.1	0.800
rs1801693	G	0.865	0.917	0.614	14.7±2.5	19.4±4	1±4.7	5.1E-03
rs7761293	A	0.763	0.838	0.381	7.7±2	4.2±3	9.7±4.4	0.541
rs6415084	T	0.427	0.429	0.5	13.8±1.7	11.9±2.1	14.9±4	0.755
rs6922216	G	0.225	0.283	0	16.5±2	13.9±2.3	12.9±6.7	0.652
rs10455872	G	0.011	0.001	0.024	91.7±7.5	85.5±39.1	116.8±9.9	0.113
rs7755463	T	0.364	0.45	0	11.9±1.7	10.7±2.1	13.2±5.8	0.440
rs6926458	A	0.892	0.915	0.833	14.2±2.7	14.6±4	9.2±5.5	0.306
rs6930542	C	0.183	0.234	0	25.9±2.1	20.3±2.5	25.4±7.5	0.360
rs9457951	G	0.192	0.249	0	25.4±2.2	20.2±2.5	29±7.8	0.180
rs1321196	C	0.447	0.482	0.321	9.7±1.7	11.1±2.1	2.2±4	0.188
rs1652507	T	0.924	0.953	0.855	24.2±3.5	39.4±5.5	9.4±6.5	0.017
rs9346833	C	0.607	0.652	0.429	7.8±1.8	10.6±2.3	−2.6±4.2	2.8E-04

The percent change in Lp(a) level per *LPA* allele is shown with standard error for the total population (effect_all_), JHS-AFR-2*_LPA_* (effect_afr_) and JHS-EUR-1_2*_LPA_* (effect_eur_) for SNPs with p_all_<0.00085. The SNP frequencies of the allele producing higher Lp(a) levels in the total population (f_all_) are shown, along with the corresponding frequency in JHS-AFR-2*_LPA_* (f_afr_) and JHS-EUR-2*_LPA_* (f_eur_). The p-value for significance of interaction of genotype with local ancestry (p_int) is also shown.

### Common *LPA* variants explain 7% of the variability in Lp(a) levels

To determine if a multi-SNP model could better explain the variability in Lp(a) levels, we performed stepwise linear regression combined with ANOVA (see Methods). Such analyses are prone to overestimation if SNP discovery, model building (SNP selection and parameter estimation) and variance calculations are all performed using the same individuals. We thus undertook five-fold cross-validation to more accurately estimate the percentage of Lp(a) variance explained by common *LPA* gene polymorphisms (see Methods). Using this approach, we found that common *LPA* variants explain 7±1% of the variance in gender-adjusted Lp(a) level. The strongest contribution was seen for rs9457951, which explains 5±1% of the variance.

Using all JHS individuals, we also built a 10 SNP model by stepwise linear regression for validation in other cohorts (see below).

### Common Variants at *LPA* Explain Most of the Association of Local Ancestry with Lp(a) Level

Many of the most highly correlated SNPs we identified are quite differentiated in frequency between JHS-AFR-2*_LPA_* and JHS-EUR-2*_LPA_*, and thus may explain the observed association of *LPA* local ancestry with Lp(a) levels (Table S1, Table S2). We tested this systematically for each SNP by comparing:

1. R^2^: the adjusted R^2^ for the regression of *LPA* local ancestry on gender-adjusted Lp(a) level
2. R^2^_geno_: the adjusted R^2^ for the regression of *LPA* local ancestry on gender- and genotype-adjusted Lp(a) level

In principle, a statistic π = 1-(R^2^/R^2^_geno_) should give the percentage of the ancestry-specific variation explained by the SNP genotype. Table S2 shows π for the 10 SNPs that most strongly account for the ancestry-specific variance. Each of these 10 SNPs has π>0.25, with 4 (rs7755463, rs9365166, rs9457951, rs225830) having π>0.40. Thus a number of individual SNPs can explain a large fraction of the observed association between *LPA* local ancestry and Lp(a) level.

Since many of these SNPs are in linkage disequilibrium, we can look for a multi-SNP model that explains a larger fraction of the ancestry. We performed stepwise-model building, repeating five-fold cross-validation, and testing the fitted model for explanation of the local ancestry contribution to Lp(a) variance. We estimate that common *LPA* variants explain 73±13% of this ancestry association.

### rs9457951 Is Strongly Associated with Isoform-adjusted Lp(a) Levels in an Independent Population

To validate our results in an independent population, we genotyped rs9457951 in 1,726 African Americans and 996 European Americans in DHS, and found a minor allele frequency of 0.176 and 0.00253 respectively. The African Americans in DHS had previously been genotyped using a panel of 2,270 genomewide ancestry informative markers (Smith et al. 2004), allowing us to generate global and local ancestry estimates. Furthermore, the kringle(IV) CNV had also been genotyped in DHS [19], allowing us to investigate the joint effects of isoform number, ancestry, and rs9457951 on Lp(a) level.

DHS African Americans had a mean of 15.8% European ancestry, with mean Lp(a) levels of 97.9 nmol/L, compared to 56.2 nmol/L in the DHS European Americans. Isoform number was a strong predictor of Lp(a) level in DHS African Americans, with a 13.9±0.4% decrease in Lp(a) per unit increase in isoform number (p = 4.2×10^−180^), accounting for 40.7% of the Lp(a) variance. We confirmed strong associations of Lp(a) with global ancestry (13±3% increase in Lp(a) per 10% increase in African ancestry proportion; p = 1.4×10^−5^) and local ancestry (9±1% increase in Lp(a) per 10% increase in African ancestry, p = 3.9×10^−13^). These estimates were reduced somewhat by adjustment for isoform number (11±2% and 7±1%, respectively), indicating a modest correlation of isoform number with global and local ancestry.

**Figure 4:**
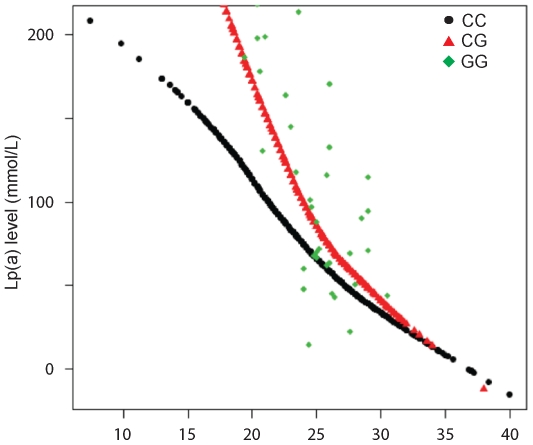
Variation of Serum Lp(a) with rs9457951 genotype in African Americans in DHS, stratified by kringle(IV) copy number. For genotypes CC and CG, the locally weighted scatterplot smoothing curve was drawn; for genotype GG, the raw data were plotted.

Genotype at rs9457951 was strongly associated with Lp(a) levels in DHS after correction for global and local ancestry (p = 3.4×10^−9^) and after additional correction for isoform number (p = 1.4×10^−24^), with a large effect on unadjusted and isoform-adjusted Lp(a) levels (29±4% and 41±3% per allele, respectively). The rs9457951 genotype was in fact able to account for 6.7% of the variance in isoform-adjusted Lp(a) levels. Figure 4 demonstrates Lp(a) levels in DHS African Americans represented according to their rs9457951 genotype and stratified by isoform number, showing a marked effect across multiple isoforms. Although the absolute change in Lp(a) level per rs9457951 allele decreases with isoform number, the percentage change is relatively constant, as evidenced by a non-significant genotype x isoform interaction on log-transformed Lp(a) levels (p = 0.14).

In addition to rs9457951, we investigated 3 SNPs that had previously been reported to explain a large proportion of African-European differences in Lp(a) level [10]. As the prior study had not incorporated estimates of global and local ancestry, we wished to analyze these SNPs with adjustment for ancestry and isoform number. Of the 3 SNPs tested (rs1801693, G+1/inKIV-8A, and T3888P (rs41272110)), rs1801693 explains 11% of the isoform- and ancestry-adjusted variance, G+1inKIV-8A explains 3.7%, and T3888P explains 32%. In a similar analysis, rs9457951 explains 36% of isoform- and ancestry-adjusted variance in Lp(a), while a combination of T3888P and rs9457951 genotypes explains 56%.

### rs9457951 is not Associated with Coronary Heart Disease Outcomes in ARIC

We evaluated the 10 SNP model of common *LPA* variants that we had discovered in JHS in 2200 African Americans from ARIC. Although there were 3225 African Americans in ARIC with Lp(a) levels and DNA available, 1000 of these are also JHS participants and had been included in the JHS analysis, so they were excluded from validation of SNP and ancestry association with Lp(a) levels in ARIC. We found strong association of local ancestry at the *LPA* locus with Lp(a) level (p<2.2×10^−16^) and confirmed association of 7 of the 10 SNPs at p<0.005 (Table S3). Furthermore, in keeping with the cross-validation results in JHS, we found that our 10 SNP model explained 71% of the association of local ancestry with Lp(a) level. However, despite these strong associations with Lp(a) level, we found no association of any of the 10 SNPs, or the 10 SNP model with the 389 incident CHD outcomes in the 3225 ARIC participants with surveillance data (Table S3). Furthermore, no significant association was seen for global (p = 0.14, β = −0.76, 95% CI −1.79–0.25) or local ancestry with CHD outcomes (p = 0.31, β = 0.12, 95% CI −0.11–0.34).

We were interested in determining whether we were adequately powered to detect an effect of genotype on myocardial infarction. Within ARIC, Lp(a) levels have weak associations with CHD outcomes, with a previously reported relative risk of 1.15 for African American women and 1.01 for African American men per unit standard deviation in Lp(a) level (∼100 mg/dL) [20]. As relative risk and hazard ratios (HR) are not readily comparable, we used the previously reported HR of 1.22 per doubling of Lp(a) level [1] observed in European populations and estimated that we would have to be powered to detect an HR of 1.07 for the corresponding ∼25% change in Lp(a) seen per allele of rs9457951. Simulation-based power estimates using the exponential distribution to model survival times demonstrated that we with the 389 outcomes in ARIC, we only had 12% power to detect an HR of 1.07 or less at p<0.05 for the rs9457951 allele (see Methods).

### Lp(a)-associated SNP Regions Harbor Potential Transcription Factor Binding Sites

**Figure 5:**
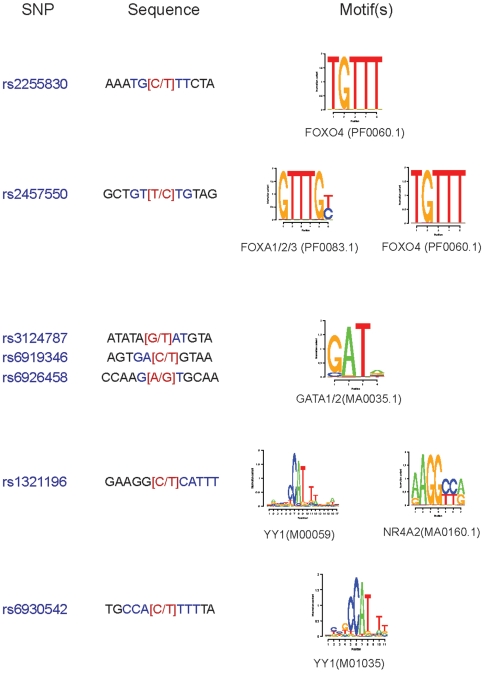
Mapping of Lp(a) associated SNP regions to transcription factor binding motifs. Sequences surrounding each of the 24 Lp(a) significantly associated SNPs were scanned for transcription factor binding motifs from the Transfac and Jaspar databases. Sequence logos for binding motifs predicted to be disrupted by allelic variation are displayed along with the HGNC symbol of associated transcription factor and the Jaspar or Transfac motif ID. Only transcription factors with literature evidence of liver expression are shown.

In addition to identifying SNPs that may contribute to disease risk, genetic association studies have the potential to illuminate the regulatory transcriptional architecture of quantitative traits. Recent studies have mapped transcription factor motifs to DNA sequences harboring genetic variants and have subsequently used chromatin immunoprecipitation to demonstrate genotype-dependent occupancy of the binding site [21]. To identify potential transcription factor binding sites that may be influenced by genetic variation at the Lp(a) locus, we scanned the genomic sequences surrounding the 24 Lp(a) associated SNPs using Jaspar (http://www.jaspar.genereg.net) and Transfac (http://www.gene-regulation.com) positional weight matrices (PWMs) with quantitative thresholds for match quality (see Methods). We focused on transcription factors with previously documented expression in liver (the site of Lp(a) production) and identified 7 SNPs for which genetic variation is likely to influence binding of such transcription factors (Figure 5). The transcription factors for the various SNPs include members of the GATA (rs3124787, rs6919346, rs6926458), and Forkhead families (rs2255830, rs2457550). Interestingly, the strongly associated rs6930542 SNP is expected to influence the binding of YY1, a ubiquitously expressed transcription factor with potential for either activating or repressive effects on gene expression [22]. In this case the C allele associated with higher Lp(a) levels and higher frequencies in African ancestral populations would be expected to disrupt YY1 binding, suggesting that the baseline transcriptional effect of YY1 at this site would be repressive. Further experiments in liver tissue and/or cell lines will be needed to validate these predictions.

## Discussion

We have used a combination of admixture mapping and fine-mapping adjusted for local ancestry to characterize the genetic basis of interethnic differences in Lp(a) levels. Given that there is a pronounced influence of global ancestry on Lp(a) levels *within* African Americans (∼10% change in Lp(a) per 10% increase in African ancestry), genetic determinants of this association might, in fact, explain much of the difference in Lp(a) levels *between* African and European American populations. Towards this end, dense fine-mapping identified common biallelic SNPs that account for >70% of the global and local ancestry signal. Furthermore, the observed, prominent peak of admixture association indicated that variants differing in frequency between the African and European ancestral populations are important determinants of Lp(a) level in African Americans. One of these variants, rs9457951, explains up to 5% of Lp(a) variance, and, in combination with additional common SNPs, accounts for a total of 7% of variance.

We replicated the association of rs9457951 in the DHS cohort and demonstrated that the effect of rs9457951 is even more marked on isoform-adjusted Lp(a) levels. Similarly, rs9457951 explains a large proportion of the *LPA* local ancestry signal in DHS. In contrast to the recently described association of biallelic variants rs3798220 and rs10455872 with Lp(a) levels in Caucasian populations [2], no single common biallelic *LPA* variant explains a large proportion of variation in Lp(a) levels in African Americans. This is most likely a reflection of limited linkage disequilibrium between extreme kringle(IV) repeat numbers and common SNPs on the African ancestral background and is in keeping with recently observed genetic architecture for the Lp(a) locus in Chinese and South-Asian populations [23]. In contrast to common biallelic SNPs, kringle(IV) copy numbers explained >40% of Lp(a) variance in DHS. Additional copy number variants and less common biallelic variants within *LPA* and other genes are expected to contribute to the remainder of the variance.

We further replicated the strong association of *LPA* local ancestry with Lp(a) levels in a third large cohort of African Americans and confirmed association of the majority of the SNPs in our multi-SNP model. However, we found no significant association of any *LPA* variant with CHD outcomes. This negative result can be attributed in part to the lower contribution of individual biallelic variants on Lp(a) levels in African Americans as compared to that seen for European Americans [14] as evidenced by our low power (12%) to detect the expected HR. It remains to be seen whether, with a much larger number of CHD cases, an association with biallelic variants in the LPA genes will be seen in African Americans.

## Methods

### Ethics Statement

This study was conducted according to the principles expressed in the Declaration of Helsinki. The study was approved by the Institutional Review Board of the Jackson Heart Study, ARIC and Dallas Heart Study. All patients provided written informed consent for the collection of samples and subsequent analysis.

### Selection of Case and Control samples

Participants in the discovery sample for this study (n = 4605) were all self-identified African Americans in the Jackson Heart Study [2]. Between Sept. 2000 and March 2004, 5,301 African Americans were recruited from three counties, Hinds, Rankin, and Madison, which comprise the Jackson, MS metropolitan area. Unrelated JHS participants were drawn from three sources in roughly equal numbers: (1) former ARIC participants; (2) participants selected randomly from a commercial residential listing; and (3) a constrained volunteer sample for which demographic cells for recruitment were designed to mirror the overall target population. 4464 individuals were successfully genotyped on the admixture panel [24], [13], [25]. DHS samples were previously described in [26]. The ARIC study is a prospective cohort study of 15,792 participants investigating the etiology of atherosclerosis and described in detail elsewhere [27].

### Lp(a) Assay

Serum Lp(a) was analyzed in JHS samples by a Diasorin nephelometric assay on a Roche Cobas FARA analyzer [28]. Lp(a) was analyzed in DHS using a sandwich ELISA that is insensitive to apo(a) isoform size [29]. Isoform number was determined in DHS by immunoblot analysis with an *LPA*-specific antibody [30]. Lp(a) was analyzed in ARIC using a double-antibody ELISA [31].

### Statistical Analysis

Linear regression analyses were used to evaluate association of ancestry with Lp(a) levels. Association analyses were performed using a combination of *MERLIN* [32] and *R* (www.Rproject.org). *MERLIN* was used to obtain p-values and regression coefficients over the entire cohort, accounting for the family structure among related individuals in JHS. For cross-validation, which was needed for accurate estimates of SNP contribution to Lp(a) variance (total and ancestry-specific), we used *R* (2.9.1), which allowed greater analytic flexibility. However, since we were unable to correct for family structure in cross-validation analyses, we randomly selected only one member of each family to be included in any given analysis. To minimize bias, we generated 100 overlapping groups of 3300 unrelated individuals and conducted all statistical analyses on each set, averaging the results. The pairwise LD plot was generated using the *LDheatmap* package in R.

### Linear Regression Models

Lp(a) values in mg/dL were log transformed. Sex-adjusted Lp(a) was used as the phenotype in association analyses with global ancestry, local ancestry, and genotype – we did not see a significant association of Lp(a) levels with age. For genotype-phenotype association analyses, we assumed an additive mode of inheritance and tested for the strength of association by ANOVA [33] with nested linear regression models, which included global ancestry, local ancestry, and local ancestry + genotype. The genotype-local ancestry interaction term was computed as a product of the local-ancestry term and SNP genotype, and is a continuous variable ranging from of 0 to 2. We conducted separate linear regression in a subset of 1831 individuals with a >95% probability of two African ancestry alleles at the *LPA* locus and in a subset of 615 individuals with a high (>95%) probability of 1 or more European ancestral alleles (local ancestry >48% European ancestry). We also identified a small subgroup of 46 individuals with >95% probability of two European ancestral alleles JHS-EUR-2*_LPA_* for calculation of allele frequencies and linkage disequilibrium parameters (Table 1). Effect sizes were estimated by evaluating what effect a unit change in genotype would have on the predicted value of the trait.

Multi-SNP models were identified with stepwise linear regression and the *anova* function in *R*. For each of the 100 sets of 3300 unrelated individuals, the top SNP associated with the phenotype residual of interest was identified and added to the regression model. The remaining SNPs were then each tested sequentially by ANOVA by comparing the model with and without the SNP and adding the SNP to the model if the p-value for comparison was <0.05. At each step this process was performed for each of the 100 sets of individuals and the average p-value for all sets was determined. The process was continued until no additional SNP improved the model at the p<0.05 threshold.

### Cross-Validation for Assessment of Percentage of Lp(a) Variance Explained

Cross validation was used for estimation of contribution of individual SNPs and a multi-SNP model to Lp(a) variance. For multi-SNP model assessment, each of the 100 groups of 3300 unrelated JHS individuals was randomly divided into 5 sets. For each group, 3 sets were used for SNP discovery (p<0.05 for association with sex and ancestry-adjusted Lp(a)), one used to build a model by stepwise regression/ANOVA (including coefficient estimation), and the fifth used as a test set to evaluate either the percentage of residual variance or local-ancestry specific variance explained. The roles of each set were rotated to obtain unbiased estimates across the entire cohort. For single-SNP assessment, two-fold cross-validation was performed, with one set used for coefficient estimation and the second for determination of the percentage of Lp(a) variance explained. The percentage of local ancestry-specific variance in Lp(a) explained was calculated as described in the Results section.

The local ancestry estimate at *LPA* was improved by forcing rs9457951 into the set of markers used to estimate local ancestry. This estimate was used for all analyses. Estimation of allele frequencies and r^2^ for SNPs was performed using the *R GeneticsBase* package.

### CHD Outcomes

We focused on 3225 African Americans in ARIC for this analysis, excluding individuals based on the following criteria; participants from centers with small numbers (n = 55), prevalent CHD (n = 139), missing data for prevalent CHD (n = 63), and missing genotype data for the respective SNPs. The final analysis sample included 389 incident CHD cases. Ascertainment and standardized case definitions for CHD have been described elsewhere [34]. Ten *LPA* SNPs were genotyped on stored DNA using the TaqMan® System. We tested for Hardy Weinberg equilibrium using the χ^2^ goodness of fit test. Cox proportional hazard regression was used to estimate the associations of SNPs and incident events and linear regression was used for associations of SNP genotype and ancestry with log-transformed Lp(a) levels. All data were analyzed with STATA, Version 10.1.

Power calculations for rs9457951 were conducted by simulation of the Cox Proportional Hazards Model in *R* as described [35]. We estimated that if the hazard ratio (HR) matched that seen in [1], which was 1.22 for a doubling of Lp(a) level, we should see an HR of 1.067 for a change in 25% in serum Lp(a), which is that seen for inheritance of each allele of rs9457951 (or rs6390542). We estimated our power to detect such an HR as follows. For each of 1000 iterations, using the estimated allele frequency in ARIC, genotypes (*z_i_*) were randomly sampled for 3225 individuals. We then simulated survival times for the *i^th^* individual were generated randomly using the exponential distribution, conditional on genotype and HR, where the HR was drawn from a normal distribution with mean = 1.067 and standard deviation (as a percentage of mean) matching that seen for age in the Cox Proportional Hazards model. A survival time threshold for each simulation was selected so that the number of CHD outcomes matched that seen in ARIC (389). The significance of the association of genotype with CHD outcome was determined using the *coxph* function in *R*. The process was repeated 1000 times and the percentage of significant associations (p<0.05) – which was found to be 11.7% - was used to estimate power.

To determine case and control samples for admixture mapping, individuals were ranked in terms of increasing gender-adjusted Lp(a) level (age was not seen as a significant covariate), and the top and bottom quintiles were used as cases and controls, respectively.

#### Admixture Mapping and Markov Chain Monte Carlo data analysis for inference of ancestry and testing of disease association

The ANCESTRYMAP software [17] was used for all analyses. The program generates local ancestry estimates by integrating information from a panel of densely spaced markers differentiated in frequency between African and European populations. The use of admixture mapping in JHS has been described previously [13], [24], [25]. Briefly, we focused on the use of a “Cases-Only” statistic, looking at regions in the genome where the local ancestry in Cases deviates significantly from the average ancestry across the genome. The control population was used to ensure that no artifact contributed to the increase in ancestry at the peak (i.e. we expect that controls should have a change in ancestry in the opposite direction). Admixture peaks were defined by regions where posterior LOD scores exceed 0. Bayesian 95% credible intervals were then computed by plotting the posterior LOD scores across the chromosome of interest and defining the region centered on the maximum LOD score that included 95% of the peak area.

### Fine-Mapping Genotyping

Genotyping was performed on the Sequenom platform, which utilizes matrix-assisted laser-desorption ionization time-of-flight mass spectroscopy. SNPs with a genotype call rate <90% (n = 12) and individuals with genotyping success rates <f85% (n = 141) were excluded from analysis. Using Tagger [36], SNPs were selected to tag the LPA gene (including 10 kb upstream and downstream) locus at an r^2^ of 1.0 with a minor allele frequency (MAF) ≥2% in the Yoruba West African HapMap population (YRI) using HapMap release #24. We then forced these SNPs into Tagger to tag this region in CEU at an r^2^ of 1.0 with MAF ≥2%.

### Prediction of Transcription Factor Binding Sites at SNP regions

Positional weight matrices (PWMs) for mammalian transcription factors (TFs) were obtained from the Jaspar [37] (http://www.jaspar.genereg.net) and Transfac [38] (http://www.gene-regulation.com) databases. To compute a probability of protein occupancy for each transcription factor at each site, a previously described method was adapted [39]. Briefly, binding of protein *X* to site *i* was modeled using the simple binding isotherm:
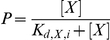
where [X] is the free concentration of protein X and K_d,X,i_ is the sequence-dependent dissociation constant for protein X at site i. Although [X] is not known in most experimental situations, it can be estimated as equal to the dissociation constant for the optimal binding site in the genome, thus leading to a probability of occupancy of 50% at optimal sites. The dissociation constant is calculated from the free energy of binding, which in turn is estimated from comparing the observed frequency *f_bj_* of base *b* at each position *j* in the PWM with the background frequency of that base *p_b_* in the genome:
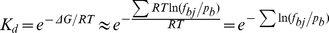



The genomic sequences 20 bases upstream and downstream of each *LPA* SNP of interest were downloaded from the UCSC Genome Browser (http://genome.ucsc.edu/). For each of the 1445 Transfac and Jaspar PWM's a maximal probability of occupancy was computed for sliding windows across the 41bp. Motifs shown in Figure 5 correspond to transcription factors with probabilities of occupancy >0.20 and a difference in probability between the two alleles of >0.20. Results were robust to probability ranges between 0.10 and 0.30. Identified transcription factors were confirmed to have previously documented expression in mammalian liver by consulting the Human Protein Reference Database [40] (http://www.hprd.org) and PubMed (http://www.pubmed.org).

## Supporting Information

Figure S1 Pairwise linkage disequilibrium measures (r^2^) for significantly associated SNPs in the JHS European local ancestry subpopulation.
(13.62 MB TIF)

Table S1 Association of LPA variants with Lp(a) levels. p-values for association of Lp(a) with genotype in the total population (p_all_), JHS-AFR-2_LPA_ (p_afr_), and JHS-EUR-1_2_LPA_ (p_eur_). SNPs with p<0.00085 are denoted by an asterisk. The SNP frequencies of the allele producing higher Lp(a) levels in the total population (f_all_) is shown, along with the corresponding frequency in JHS-AFR-2_LPA_ (f_afr_) and JHS-EUR-2_LPA_ (f_eur_). The chromosomal position in bases (NCBI Build 36) is also provided.
(0.14 MB DOC)

Table S2 Effect of LPA variants on Association of LPA local ancestry with Lp(a) levels. π, which is the fraction of ancestry-specific variation in Lp(a) levels explained by genotype, is shown for the genotyped SNPs that account for the greatest amount of ancestry specific variance. The SNP frequencies and effect sizes in the overall population are shown as in Table 2; p_all_ is shown as in Table S1.
(0.06 MB DOC)

Table S3 Effect of LPA variants on Lp(a) levels and CHD outcomes in ARIC. P-values for association of SNPs with Lp(a) levels in a linear regression model with age and gender are shown, along with coefficients, confidence interval (CI) and p-value for association with CHD outcomes in a Cox regression model. A p-value for the significance of including 10 SNP genotypes in a model to predict CHD outcomes was computed using the log-likelihood ratio test.
(0.07 MB DOC)
