# Supplementary figures and images for: Single-Nucleotide Polymorphisms in LPA Explain Most of the Ancestry-Specific Variation in Lp(a) Levels in African Americans
Source: PLoS One. 2011 Jan 24;6(1):e14581. doi: 10.1371/journal.pone.0014581 (PMC3025914; doi:10.1371/journal.pone.0014581)

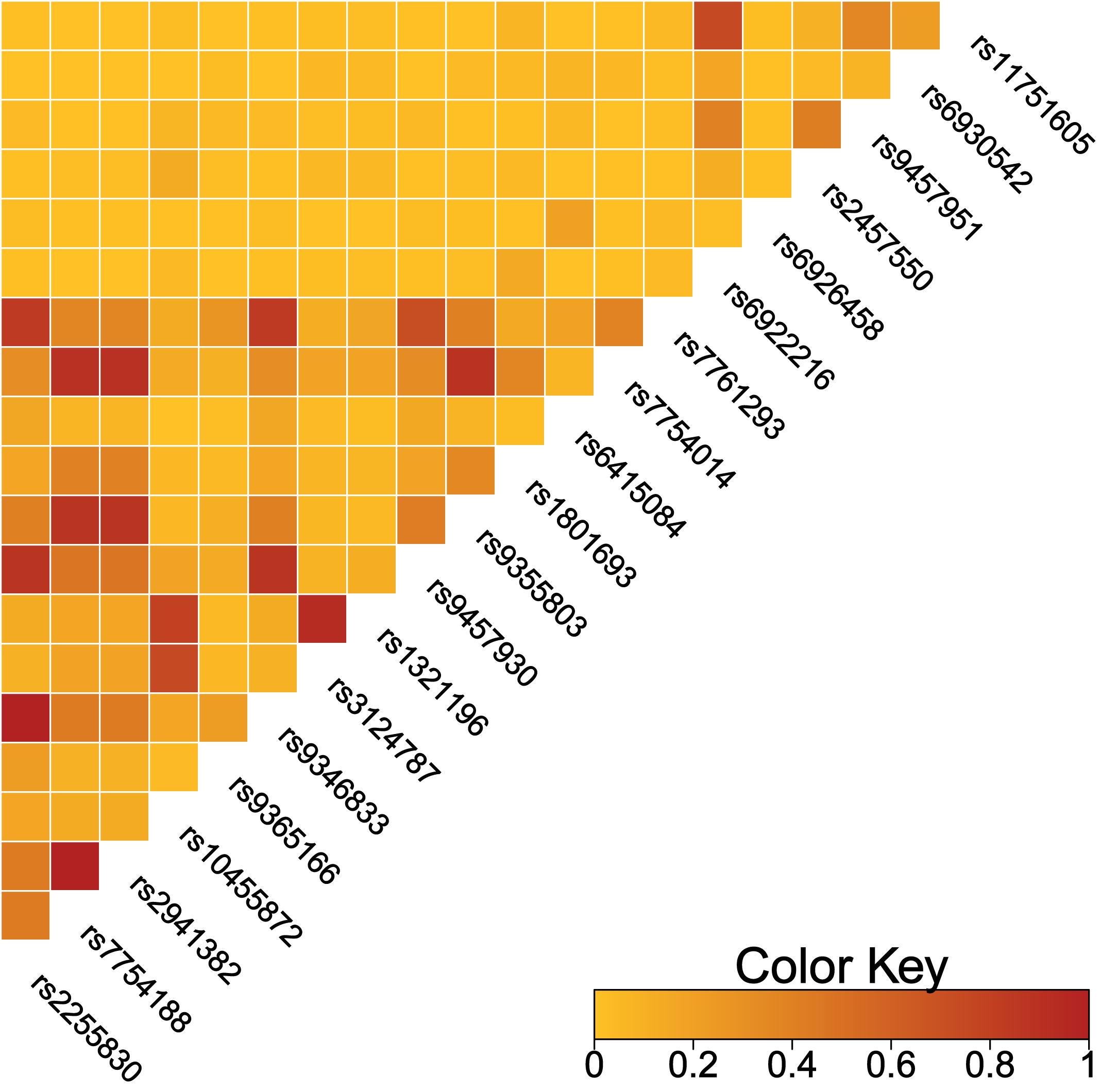

Supplement: Figure S1 — Pairwise linkage disequilibrium measures (r2) for significantly associated SNPs in the JHS European local ancestry subpopulation. (13.62 MB TIF) [file pone.0014581.s001.tif]
