# Supplementary material for: Single-Nucleotide Polymorphisms in LPA Explain Most of the Ancestry-Specific Variation in Lp(a) Levels in African Americans
Source: PLoS One. 2011 Jan 24;6(1):e14581. doi: 10.1371/journal.pone.0014581 (PMC3025914; doi:10.1371/journal.pone.0014581)
Supplement: Table S1 — Association of LPA variants with Lp(a) levels. p-values for association of Lp(a) with genotype in the total population (pall), JHS-AFR-2LPA (pafr), and JHS-EUR-1_2LPA (peur). SNPs with p<0.00085 are denoted by an asterisk. The SNP frequencies of the allele producing higher Lp(a) levels in the total population (fall) is shown, along with the corresponding frequency in JHS-AFR-2LPA (fafr) and JHS-EUR-2LPA (feur). The chromosomal position in bases (NCBI Build 36) is also provided. (0.14 MB DOC) [file pone.0014581.s002.docx]

| SNP | f_all_ | f_afr_ | f_eur_ | p_all_ | p_afr_ | p_eur_ | Position |
| --- | --- | --- | --- | --- | --- | --- | --- |
| rs2504921 | 0.579 | 0.62 | 0.356 | 0.0038 | 2.70E-04 | 0.17 | 160864417 |
| rs2255830* | 0.776 | 0.847 | 0.422 | 2.20E-11 | 2.50E-07 | 0.15 | 160864776 |
| rs2457550* | 0.872 | 0.918 | 0.727 | 2.70E-07 | 4.00E-05 | 0.78 | 160865540 |
| rs2136675* | 0.861 | 0.906 | 0.625 | 2.80E-06 | 5.50E-05 | 0.94 | 160866842 |
| rs2457548 | 0.655 | 0.613 | 0.864 | 0.02 | 0.086 | 0.2 | 160869052 |
| rs9346817 | 0.925 | 0.95 | 0.8 | 0.0019 | 0.06 | 0.054 | 160872680 |
| rs9355803* | 0.927 | 0.949 | 0.814 | 7.20E-04 | 0.03 | 0.064 | 160873589 |
| rs3123629 | 0.278 | 0.284 | 0.267 | 0.063 | 0.86 | 0.026 | 160876497 |
| rs2941382* | 0.95 | 0.976 | 0.856 | 4.00E-04 | 0.22 | 0.0109 | 160876851 |
| rs9347407 | 0.629 | 0.678 | 0.407 | 0.143 | 0.84 | 0.25 | 160882093 |
| rs9365166* | 0.782 | 0.853 | 0.422 | 3.60E-12 | 3.00E-08 | 0.134 | 160882319 |
| rs9346818 | 0.581 | 0.626 | 0.352 | 0.002 | 1.10E-04 | 0.062 | 160884871 |
| rs7754014* | 0.444 | 0.495 | 0.233 | 5.80E-05 | 1.80E-04 | 0.079 | 160888706 |
| rs7754188* | 0.305 | 0.325 | 0.222 | 1.50E-09 | 8.3E-09 | 0.97 | 160888825 |
| rs7759553 | 0.047 | 0.055 | 0 | 0.57 | 0.86 | 0.84 | 160889782 |
| rs9457930* | 0.795 | 0.869 | 0.439 | 2.50E-10 | 8.20E-06 | 0.121 | 160891977 |
| rs10945674 | 0.777 | 0.787 | 0.733 | 0.28 | 0.024 | 0.057 | 160897331 |
| rs9364558 | 0.626 | 0.647 | 0.534 | 0.17 | 0.0135 | 0.24 | 160900105 |
| rs3127595 | 0.459 | 0.425 | 0.622 | 0.027 | 0.007 | 0.91 | 160909638 |
| rs7453836 | 0.433 | 0.442 | 0.398 | 0.0072 | 0.0099 | 0.92 | 160913302 |
| rs3124787* | 0.866 | 0.918 | 0.6 | 6.30E-06 | 1.50E-04 | 0.91 | 160916860 |
| rs3127596 | 0.716 | 0.709 | 0.739 | 0.6 | 0.03 | 0.066 | 160923446 |
| rs7449940 | 0.961 | 0.957 | 1 | 0.002 | 0.115 | 0.45 | 160925259 |
| rs9457933 | 0.894 | 0.876 | 0.952 | 0.96 | 0.81 | 0.51 | 160928180 |
| rs6922557 | 0.975 | 0.971 | 1 | 0.23 | 0.41 | 0.018 | 160928995 |
| rs6919346* | 0.965 | 0.994 | 0.81 | 1.40E-07 | 0.03 | 0.0024 | 160930770 |
| rs7767084 | 0.968 | 0.997 | 0.869 | 0.016 | 0.59 | 0.26 | 160932664 |
| rs11751605* | 0.024 | 0.001 | 0.107 | 1.80E-04 | 0.057 | 6.4E-05 | 160933391 |
| rs1801693* | 0.865 | 0.917 | 0.614 | 4.30E-08 | 7.30E-06 | 0.83 | 160940040 |
| rs7761293* | 0.763 | 0.838 | 0.381 | 2.30E-04 | 0.17 | 0.031 | 160941374 |
| rs9364559 | 0.927 | 0.956 | 0.786 | 0.0022 | 0.13 | 0.109 | 160946559 |
| rs12179431 | 0.835 | 0.84 | 0.856 | 0.037 | 0.32 | 0.32 | 160946610 |
| rs6415084* | 0.427 | 0.429 | 0.5 | 1.50E-14 | 1.50E-07 | 3.5E-04 | 160950741 |
| rs9365171 | 0.559 | 0.523 | 0.69 | 0.24 | 0.34 | 0.053 | 160952147 |
| rs7765781 | 0.41 | 0.341 | 0.69 | 0.051 | 0.064 | 0.19 | 160977907 |
| rs7765803 | 0.555 | 0.616 | 0.318 | 0.53 | 0.55 | 0.052 | 160977949 |
| rs6922216* | 0.225 | 0.283 | 0 | 5.40E-15 | 2.00E-08 | 0.063 | 160979532 |
| rs10455872* | 0.011 | 0.001 | 0.024 | 1.30E-19 | 0.061 | 1.7E-16 | 160980279 |
| rs7755463* | 0.364 | 0.45 | 0 | 8.90E-11 | 2.00E-06 | 0.026 | 160982681 |
| rs7754526 | 0.866 | 0.841 | 1 | 0.73 | 0.67 | 0.36 | 160982854 |
| rs12182517 | 0.956 | 0.953 | 1 | 0.18 | 0.17 | 0.58 | 160986121 |
| rs6921912 | 0.979 | 0.975 | 1 | 0.029 | 0.053 | 0.26 | 160986402 |
| rs6913833 | 0.496 | 0.541 | 0.322 | 0.046 | 0.103 | 0.5 | 160987612 |
| rs7770628 | 0.246 | 0.2 | 0.512 | 0.137 | 0.53 | 0.0124 | 160988585 |
| rs7771129 | 0.497 | 0.541 | 0.322 | 0.074 | 0.113 | 0.39 | 160988875 |
| rs6926458* | 0.892 | 0.915 | 0.833 | 1.10E-06 | 4.60E-04 | 0.104 | 160990277 |
| rs7743535 | 0.846 | 0.816 | 1 | 0.115 | 0.114 | 0.95 | 160990799 |
| rs7450261 | 0.941 | 0.925 | 1 | 0.0039 | 0.018 | 0.25 | 160990916 |
| rs6930542* | 0.183 | 0.234 | 0 | 9.20E-27 | 1.9E-13 | 0.0017 | 160995958 |
| rs6932014 | 0.537 | 0.588 | 0.322 | 0.49 | 0.48 | 0.4 | 160996926 |
| rs9457951* | 0.192 | 0.249 | 0 | 9.20E-26 | 3.3E-13 | 7.4E-04 | 160998239 |
| rs9355813 | 0.49 | 0.533 | 0.305 | 0.016 | 0.018 | 0.48 | 161001583 |
| rs10945682 | 0.574 | 0.636 | 0.31 | 8.60E-04 | 0.0027 | 0.97 | 161040102 |
| rs1321196* | 0.447 | 0.482 | 0.321 | 2.80E-08 | 7.90E-07 | 0.58 | 161052003 |
| rs1652507* | 0.924 | 0.953 | 0.855 | 1.60E-10 | 6.5E-10 | 0.15 | 161052622 |
| rs9346833* | 0.607 | 0.652 | 0.429 | 2.80E-05 | 1.60E-05 | 0.53 | 161054803 |
| rs1358754 | 0.129 | 0.141 | 0.146 | 0.005 | 0.027 | 0.94 | 161060681 |
| rs1358753 | 0.799 | 0.775 | 0.845 | 0.102 | 0.138 | 0.075 | 161060731 |
| rs11969451 | 0.876 | 0.851 | 1 | 0.75 | 0.26 | 0.68 | 161065129 |
