# Supplementary material for: Single-Nucleotide Polymorphisms in LPA Explain Most of the Ancestry-Specific Variation in Lp(a) Levels in African Americans
Source: PLoS One. 2011 Jan 24;6(1):e14581. doi: 10.1371/journal.pone.0014581 (PMC3025914; doi:10.1371/journal.pone.0014581)
Supplement: Table S2 — Effect of LPA variants on Association of LPA local ancestry with Lp(a) levels. π, which is the fraction of ancestry-specific variation in Lp(a) levels explained by genotype, is shown for the genotyped SNPs that account for the greatest amount of ancestry specific variance. The SNP frequencies and effect sizes in the overall population are shown as in Table 2; pall is shown as in Table S1. (0.06 MB DOC) [file pone.0014581.s003.docx]

| SNP | f_all_ | f_afr_ | f_eur_ | π | p_all_ | effect_all_  ± se_all_ |
| --- | --- | --- | --- | --- | --- | --- |
| rs2255830 | 0.776 | 0.847 | 0.422 | 0.396 | 2.20E-11 | 14.5±2.0 |
| rs9365166 | 0.782 | 0.853 | 0.422 | 0.438 | 3.60E-12 | 15.1±2.0 |
| rs9457930 | 0.795 | 0.869 | 0.439 | 0.413 | 2.50E-10 | 14.3±2.1 |
| rs3124787 | 0.866 | 0.918 | 0.6 | 0.316 | 6.30E-06 | 12.3±2.6 |
| rs6919346 | 0.965 | 0.994 | 0.81 | 0.341 | 1.40E-07 | 27.5±4.7 |
| rs7761293 | 0.763 | 0.838 | 0.381 | 0.273 | 2.30E-04 | 7.7±2.0 |
| rs6922216 | 0.225 | 0.283 | 0 | 0.260 | 5.40E-15 | 16.5±2.0 |
| rs7755463 | 0.364 | 0.45 | 0 | 0.453 | 8.90E-11 | 11.9±1.7 |
| rs6930542 | 0.183 | 0.234 | 0 | 0.331 | 9.20E-27 | 25.9±2.1 |
| rs9457951 | 0.192 | 0.249 | 0 | 0.419 | 9.20E-26 | 25.4±2.2 |
