# Supplementary material for: Single-Nucleotide Polymorphisms in LPA Explain Most of the Ancestry-Specific Variation in Lp(a) Levels in African Americans
Source: PLoS One. 2011 Jan 24;6(1):e14581. doi: 10.1371/journal.pone.0014581 (PMC3025914; doi:10.1371/journal.pone.0014581)
Supplement: Table S3 — Effect of LPA variants on Lp(a) levels and CHD outcomes in ARIC. P-values for association of SNPs with Lp(a) levels in a linear regression model with age and gender are shown, along with coefficients, confidence interval (CI) and p-value for association with CHD outcomes in a Cox regression model. A p-value for the significance of including 10 SNP genotypes in a model to predict CHD outcomes was computed using the log-likelihood ratio test. (0.07 MB DOC) [file pone.0014581.s004.docx]

**Table S3: Effect of *LPA* variants on Lp(a) levels and CHD outcomes in ARIC**. P-values for association of SNPs with Lp(a) levels in a linear regression model with age and gender are shown, along with coefficients, confidence interval (CI) and p-value for association with CHD outcomes in a Cox regression model. A p-value for the significance of including 10 SNP genotypes in a model to predict CHD outcomes was computed using the log-likelihood ratio test.

| SNP | p-value (Lp(a)) | coeff (CHD) | CI (CHD) | p-value (CHD) |
| --- | --- | --- | --- | --- |
| rs7754188 | 3.99E-07 | -0.023 | -0.18 – 0.14 | 0.77 |
| rs6919346 | 2.31E-10 | -0.14 | -0.12 – 0.35 | 0.54 |
| rs2255830 | 5.28E-16 | -0.15 | -0.34 – 0.035 | 0.11 |
| rs9355803 | 0.695 | -0.19 | -0.48 – 0.10 | 0.22 |
| rs6926458 | 2.66E-09 | -0.052 | -0.30 – 0.20 | 0.68 |
| rs2941382 | 0.481 | -0.023 | -0.34 – 0.29 | 0.89 |
| rs9457951 | 5.28E-16 | 0.062 | -0.12 – 0.25 | 0.52 |
| rs10455872 | 3.57E-11 | 0.092 | -0.51 – 0.70 | 0.77 |
| rs1652507 | 4.16E-12 | -0.047 | -0.33 – 0.23 | 0.74 |
| rs7449940 | 0.264 | -0.35 | -0.81 – 0.10 | 0.13 |
| 10 SNP model |  |  |  | 0.28 |
